# Estimation of export cutoff productivity of Chinese industrial enterprises

**DOI:** 10.1371/journal.pone.0277842

**Published:** 2022-11-29

**Authors:** Lianjie Duan

**Affiliations:** School of Tourism & Business, Guangzhou Panyu Polytechnic, Guangzhou, Guangdong, China; University of Oklahama Norman Campus: The University of Oklahoma, UNITED STATES

## Abstract

Based on China’s transaction-level trade data and firm-level production data during the period 2000–2006, this paper firstly estimates the export cutoff productivity by applying non-parametric ROC method. The results are as follows. First, under the full sample, the export cutoff productivity is 7.051. Second, the export cutoff productivity of home enterprises is relatively low, and that of foreign enterprises is relatively high. Third, the export thresholds of capital-intensive and technology-intensive industries are relatively high, while that of labor-intensive industry is relatively low. Fourth, the export threshold of western provinces is relatively high, followed by central provinces and eastern provinces. In addition, this paper investigates the dynamic change of export threshold. The result indicates that the evolution of export threshold is generally a horizontal S-shape during the sample period, and after China’s accession to WTO in 2001, it is an inverted U-shape.

## 1 Introduction

Based on Krugman’s [1] model, Melitz [2] constructs a seminal international trade model with heterogeneous firms. This model indicates that the exporting of micro-enterprises shows the productivity threshold effect. Specially speaking, only the firms whose productivity is above export cutoff productivity can engage in export trade, while the firms whose productivity is between the domestic cutoff productivity and the export cutoff productivity can only operate in the domestic market, and the firms with a lower productivity will have to be eliminated from the market. However, since Melitz [2], the literature on empirical estimation of export cutoff productivity has been lacking for a long time, which not only makes us unable to know the quantitative level of export cutoff productivity, but also limits the research on export behavior of micro-enterprises to a certain extent. The main reason for this defect is the lack of an effective method to estimate firms’ export cutoff productivity. Neither of the existing threshold estimation methods, including Chan [3], Hansen [4, 5], Caner and Hansen [6], Seo and Linton [7] and Gao et al. [8], is applicable to the situation in which the dependent variables are dummy variables. Therefore, it is impossible to effectively estimate the export cutoff productivity using these estimation methods.

However, fortunately, the non-parametric ROC (receiver operating characteristic) method provides a way to estimate export cutoff productivity effectively. At present, this method is widely used in medicine, engineering and natural science. Although it is also involved in economics and management, its application is basically limited to the evaluation of classification performance of prediction models, such as Buckinx and Poel [9], Banasik and Crook [10], Verbeke et al. [11], Blanco et al. [12], Cubiles-De-La-Vega et al. [13], Sever [14], and Lahiri and Yang [15]. As for other applications of this method in economics and management, only Costa et al. [16] investigate the firms’ distance from export threshold using the data covering 50% of Italian manufacturing enterprises in 2014. In fact, Costa et al. [16] construct three models estimating export threshold: (1) A pure sales model; (2) A pure productivity model; (3) A composite model. It argues that there are many factors affecting firms’ export threshold and hence using the pure models is inappropriate. So, Costa et al. [16] finally choose the composite model to examine firms’ distance from export threshold. The composite model takes productivity, size (or sales), number of workers, age and consumption of fixed capital into account. However, this paper thinks that this doing is inappropriate and even wrong. The main reason is that although there are many factors affecting firms’ export behavior, there are no relevant trade theories indicating that there exist the export thresholds related to firms’ size, firms’ age, etc. In fact, only the new new trade theory represented by Melitz [2] clearly points out that the firms’ exporting shows a threshold effect, that is, the productivity threshold effect. Therefore, this paper thinks that the composite export threshold presented by Costa et al. [16] lacks of a sufficient theoretical support, and using the composite model for study is not convincing. This is the motivation for writing this paper. Specifically, this paper uses the pure productivity model to estimate export cutoff productivity of Chinese industrial enterprises. In addition, it should be noted that the focus of this paper is different from that of Costa et al. [16]. This paper is a direct estimation of export threshold, while Costa et al. [16] focuses on the firms’ distance from export threshold.

However, when using ROC method to estimate export cutoff productivity, an attention must be paid to the possible loss of classification performance caused by the “productivity paradox”. This term refers to a phenomenon in which the productivity of exporting enterprises is actually lower than that of non-exporting enterprises. Many empirical studies have demonstrated the “productivity paradox” of Chinese enterprises, such as Lu [17], Lu et al. [18], Gao and Yin [19], Yang and He [20], and Dai et al. [21]. At present, the mainstream explanation for the cause of the productivity paradox is that there are a large number of enterprises engaging in processing trade in China [19, 21]. Yu [22] indicates that the processing trade is the most important export mode in China. Due to the generally low productivity of processing trade enterprises, the average productivity level of China’s export enterprises is lowered, which thus makes the average productivity level of export firms in China remain lower than that of non-export firms operating in domestic market. It is well known that the firms’ engaging in processing trade mainly depends on low labor cost rather than their productivity level. Therefore, it is meaningless to estimate the export cutoff productivity of these enterprises. In addition, including these enterprises in analysis will surely limit the classification performance of export cutoff productivity under the full sample. In view of this, the pure processing trade enterprises are removed from the sample. The further explanation for this is seen in note of Fig 2.

The contributions of this paper are as follows. First, this study is the first paper focusing on a direct estimation of export cutoff productivity, which significantly broaden the research on firms’ export behavior. Second, it explores the ownership, industry, region and time heterogeneity of export cutoff productivity.

The remainder of the study is organized as follows. Section 2 is the introduction of estimation method and variables. Section 3 is the data source and cleaning. Section 4 presents the estimation results, including benchmark results and heterogeneity analysis. Section 5 focuses on the evolution of export threshold. Section 6 concludes.

## 2 Estimation method and variables

### 2.1 ROC method

According to Melitz [2], the cutoff export productivity is essentially the productivity boundary between exporting firms and non-exporting firms. It can also be said to the minimum productivity level required for firms to engage in exporting. This means that when the productivity of a firm is above the export cutoff productivity, it is an exporting firm, and otherwise, it is a non-exporting firm. The ROC method is a non-parametric statistical technique that effectively estimates the optimal boundary of test variable. To conduct the ROC analysis, the export dummy *exdum* should be taken as the state variable and the total factor productivity *tfp* should be taken as the test variable. The optimal boundary, i.e. the export cutoff productivity, is defined by using Youden’s [23] J statistic that is also known as Youden index. The export threshold that maximizes the Youden index is the optimal export threshold, i.e. the export cutoff productivity. Thus, the ROC method is the simple and effective one for estimating the export cutoff productivity. A more detailed introduction to this approach is shown below.

#### 2.1.1 Confusion matrix and main evaluation indicators

In Table 1, P is the total number of positives and N is the total number of negatives. So, P+N represents the whole sample. In this paper, P is the total number of exporters and N is the total number of non-exporters. P′ is the total number of hypothesized positives and N′ is the total number of hypothesized negatives. In this paper, P′ is the total number of hypothesized exporters and N′ is the total number of hypothesized non-exporters. The true positives are the correctly classified exporters and the number of them is denoted as *TP*. The false positives are the incorrectly classified non-exporters and the number of them is denoted as *FP*. The false negatives are the incorrectly classified exporters and the number of them is denoted as *FN*. The true negatives are the correctly classified non-exporters and the number of them is denoted as *TN*.

**Table 1:** Confusion matrix.

		True Class	
		P	N
Hypothesized	P′	True Positives	False Positives
Class	N′	False Negatives	True Negatives

Note: Table 1 is based on Fawcett [24].

Based on the confusion matrix, the main evaluation indicators used to measure classification performance of export threshold are shown in Table 2.

**Table 2:** Main evaluation indicators in ROC analysis.

Indicator	Calculation	Description
Sensitivity	$TPR=\frac{TP}{P}=\frac{TP}{TP+FN}$	Ratio of correctly classified exporters to total exporters
Specificity	$TNR=\frac{TN}{N}=\frac{TN}{TN+FP}$	Ratio of correctly classified non-exporters to total non-exporters
Miss rate	$FNR=\frac{FN}{P}=\frac{FN}{FN+TP}=1-TPR$	Ratio of incorrectly classified exporters to total exporters
Fallout	$FPR=\frac{FP}{N}=\frac{FP}{FP+TN}=1-TNR$	Ratio of incorrectly classified non-exporters to total non-exporters
Accuracy	$ACC=\frac{TP+TN}{P+N}$	Ratio of correctly classified exporters and non-exporters to total exporters and non-exporters

Note: Table 2 is based on Fawcett [24] and Powers [25].

#### 2.1.2 ROC curve

The ROC curve is the trajectory of combination points of sensitivity and specificity. The horizontal axis represents specificity and the vertical axis represents sensitivity (Sometimes, the horizontal axis is 1-specificity, i.e. fallout.). Each point on ROC curve corresponds to a cutoff value that is actually some value of test variable. In this paper, the cutoff value is some value of total factor productivity *tfp*. Among a series of cutoff values, the optimal export threshold, i.e. export cutoff productivity, is the one that maximizes the Youden index.

The formula for calculating the Youden index is

J=TPR+TNR−1 (1)

where the Youden index = sensitivity + specificity—1, which indicates that the optimal export threshold or the export cutoff productivity is the one that maximizes the sum of sensitivity and specificity.

**Fig 1:**
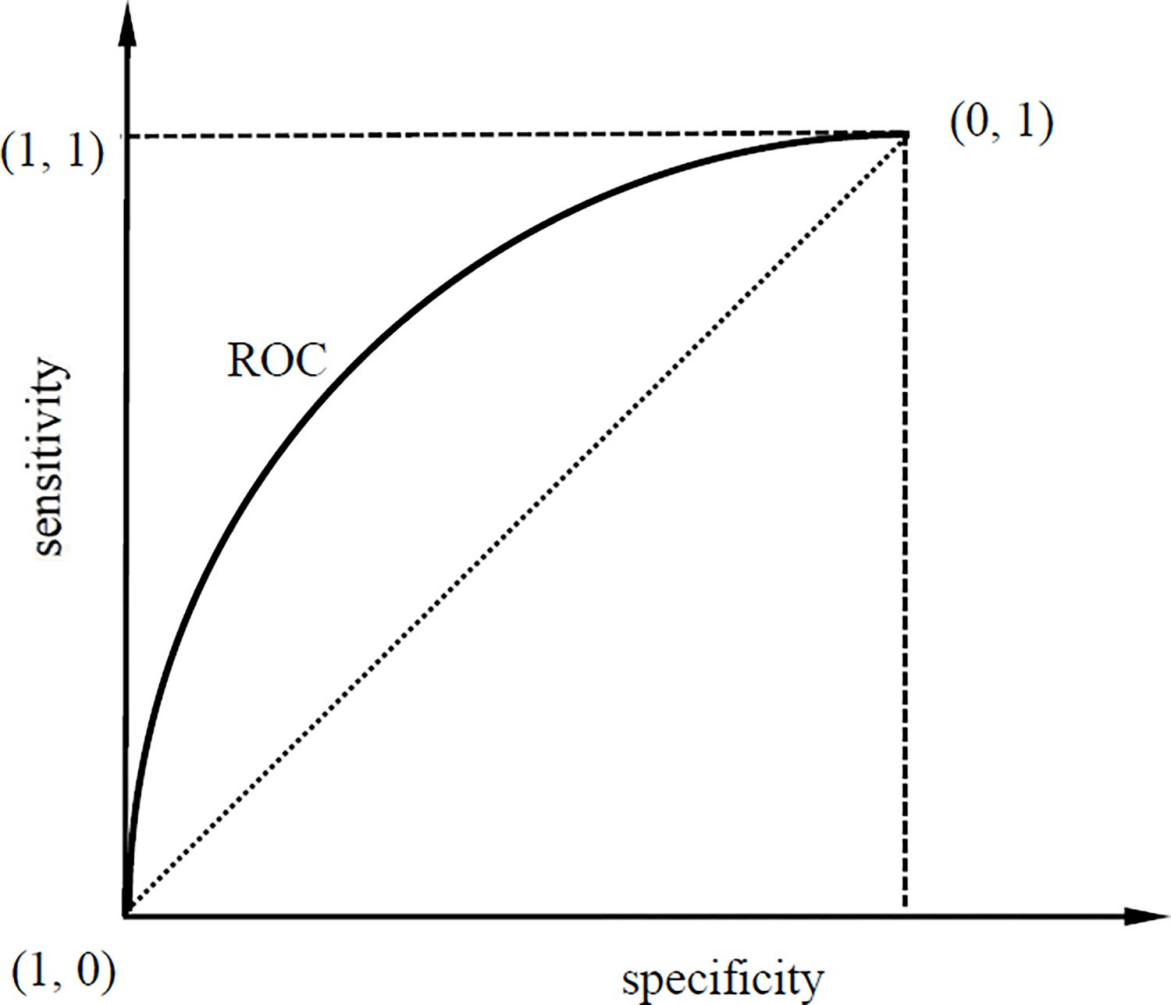
ROC curve.

In Fig 1, the horizontal axis represents specificity and the vertical axis represents sensitivity. Fig 1 indicates that specificity decreases with the increase of sensitivity. So, there exists a cutoff point that maximizes the sum of sensitivity and specificity, and this cutoff point is the optimal export threshold, i.e. the export cutoff productivity.

The area under an ROC curve is denoted as AUC, and it is the most commonly used indicator for evaluating the prediction capability of ROC curve. The bigger the AUC, the higher the classification performance. If the AUC is above 0.9, the model has the high prediction capability. If the AUC is between 0.7 and 0.9, the model has the medium prediction capability. If the AUC is between 0.5 and 0.7, the model has the weak prediction capability.

### 2.2 Variables

#### 2.2.1 Total factor productivity

Based on Olley and Pakes [26], Levinsohn and Petrin [27] propose a new semi-parametric regression method to estimate firms’ total factor productivity. The main advantage of this method is that it can effectively eliminate endogeneity and selection bias in OLS regression. LP method takes the intermediate input as the proxy variable, which is different from OP method that uses the investment as the proxy variable. However, from the availability of data, the intermediate input is easier to obtain. Further, Levinsohn and Petrin [27] provide several methods to test the desirability of proxy variables, thereby greatly expanding the selection range of them. So, LP method allows the researchers to choose the proxy variable flexibly. The specific regression equations are

$$y_{t}=\beta_{0}+\beta_{l}l_{t}+\beta_{k}k_{t}+\omega_{t}+\eta_{t}=\beta_{l}l_{t}+\phi_{t}(k_{t},\;m_{t})+\eta_{t}$$ (2)

$$\phi_{t}(k_{t},\;m_{t})=\beta_{0}+\beta_{k}k_{t}+\omega_{t}(k_{t},\;m_{t})$$ (3)

where *y*_*t*_ is the real industrial value-added of a firm, and obtained by deflating the nominal industrial value-added using the producer price index (2000 = 100). *l*_*t*_ is the labor input, and measured by the number of employees. *k*_*t*_ is the real capital input, and obtained by deflating the nominal net fixed-asset balance using the price index of fixed-asset investment (2000 = 100). *m*_*t*_ is the real industrial intermediate input, and obtained by deflating the nominal industrial intermediate input using the producer price index (2000 = 100). In the regression process, *y*_*t*_, *k*_*t*_, *l*_*t*_ and *m*_*t*_ all use the logarithmic values. All the price indexes are sourced from the official website of National Bureau of Statistics of China.

#### 2.2.2 Export dummy

*exdum*_*it*_ is the export dummy of firm *i*. If the export delivery value is greater than 0, *exdum*_*it*_ equals to 1, and otherwise, 0. In addition, one thing to be explained is that the sample used in this paper includes all the merged export and non-export enterprises, as well as the remaining non-export enterprises from Chinese Industrial Enterprises Database (CIED). For the merged enterprises, their export status can be judged not only by export delivery value in Chinese Industrial Enterprises Database, but also by export value in transaction-level trade data-set sourced from China’s General Administration of Customs (CGAC). However, if the export status of the merged firms is judged by export value of CGAC, the identification criteria of export status of all the firms in sample are not uniform because the export status of the remaining non-export firms from CIED is judged only by export delivery value. Furthermore, the total factor productivity is calculated according to CIED. Thus, if the export status of a part of firms is judged according to CGAC, the measurement basis of *tfp* and *exdum* will be inconsistent. In addition, there is no evidence indicating that the data quality of CGAC is better than that of CIED. In view of the above reasons, this paper uniformly uses the export delivery value of CIED to identify the export status of all the firms in sample.

## 3 Data

This study is based on two sets of highly disaggregated micro-enterprise data. The first set of data is the firm-level production data from Chinese Industrial Enterprises Database (CIED) obtained from China’s National Bureau of Statistics. The second set of data is the transaction-level trade data obtained from China’s General Administration of Customs (CGAC). This study is based on the matching of these two sets of data. When the existing literature uses the matching data to study the export behavior of micro-enterprises, the sample period is generally from 2000 to 2006 [21, 22, 28–32]. Following the usual practice of existing literature and considering the availability of data, this paper also uses the matching data during this period to conduct a research. Next, the processing and matching of these two sets of data is described in detail.

Chinese Industrial Enterprises Database (CIED) covers all state-owned industrial enterprises and non-state-owned industrial enterprises whose main business income exceeds RMB 5 million. The indicators include the industrial value-added, the export delivery value, the annual average number of employees, the total wages payable, and the industry category. This provides sufficient information for this study. Using the method of Brandt et al. [33], this paper integrates the first group of data. Additionally, China issued a new Classification of National Economic Industries in 2002, and officially implemented it in 2003. For the unification of coding, the industrial codes used for enterprises during the period 2000–2002 are adjusted according to the new industry classification standard. Besides, considering that there are some statistical errors in the raw data, the unreasonable observations are deleted according to the following standards: (1) The export delivery value is lower than 0; (2) The number of employees is less than 10; (3) Any of industrial value-added, industrial intermediate inputs, annual average balance of net fixed assets, firms’ age, and total wages payable is lower than or equal to 0; (4) The annual average balance of net fixed assets is higher than that of total fixed assets.

CGAC database records every import and export transaction information of enterprises. The information includes the tax number, the HS 8-digit code, the quantity, the value, the destination, the transport mode, etc. More importantly, the customs records the trade modes, including the ordinary trade, the processing trade and the other types of trade. According to the trade modes, the export enterprises can be divided into five types: (1) The pure ordinary trade enterprises; (2) The pure processing trade enterprises; (3) The enterprises purely engaging in some other type(s) of trade; (4) The mixed-enterprises at least engaging in ordinary trade; (5) The other enterprises engaging in both processing trade and some other type(s) of trade. As mentioned above, the existence of large amount of processing trade firms is the main cause for the productivity paradox, which means that the productivity is not the determinant factor of their export participation. Therefore, it is meaningless to estimate the export cutoff productivity of these enterprises and meanwhile, the classification performance of export cutoff productivity under the whole sample will be reduced if these processing trade enterprises are included in analysis. In view of this, this paper deletes the pure processing trade enterprises from the sample. In addition, considering a small proportion of other types of trade (e.g., in 2006, only 4.38%, less than 5%), this paper further removes the third and fifth types of enterprises. Therefore, after the above deleting, only the pure ordinary trade enterprises (i.e. (1)) and the mixed-enterprises at least engaging in ordinary trade (i. e. (4)) are saved.

Since the aim of this study is to estimate export cutoff productivity of enterprises under a framework of general trade, it is necessary to merge the firm-level production data that can be used to calculate total factor productivity and the transaction-level trade data that can be used to identify trade mode. This study merges the two groups of data following the method of Dai et al. [21]. Firstly, this paper matches the same enterprises between two groups with firms’ name. Further, this paper uses the postcode and the last 7 digits of telephone number to identify the same enterprises. Finally, the sample used for analysis includes all the firms that can be merged, as well as all the remaining non-export firms from Chinese Industrial Enterprises Database. The sample contains 592112 observations, of which 52423 are successfully matched, and the yearly number of successfully matched firms is 4534, 5609, 6490, 8084, 12533, 15173, respectively. In addition, the yearly number of successfully matched export firms is 2059, 2545, 2942, 3724, 5607, 6751, respectively. Due to the lack of key indicators for measuring total factor productivity, the observations in 2004 can not be applied to the subsequent estimation, and thus the result of data matching of this year is not reported. The interested readers can ask the author for it. The reason why the remaining enterprises in transaction-level trade data are not included in the sample is that this part of enterprises can not be used as the effective observations, because the calculation of total factor productivity has to rely on the firm-level production data. Meanwhile, the remaining export firms from Chinese Industrial Enterprises Database are not included because the trade modes of these firms cannot be identified.

## 4 Estimation results

### 4.1 Benchmark estimation and robustness tests

#### 4.1.1 Benchmark estimation

**Fig 2:**
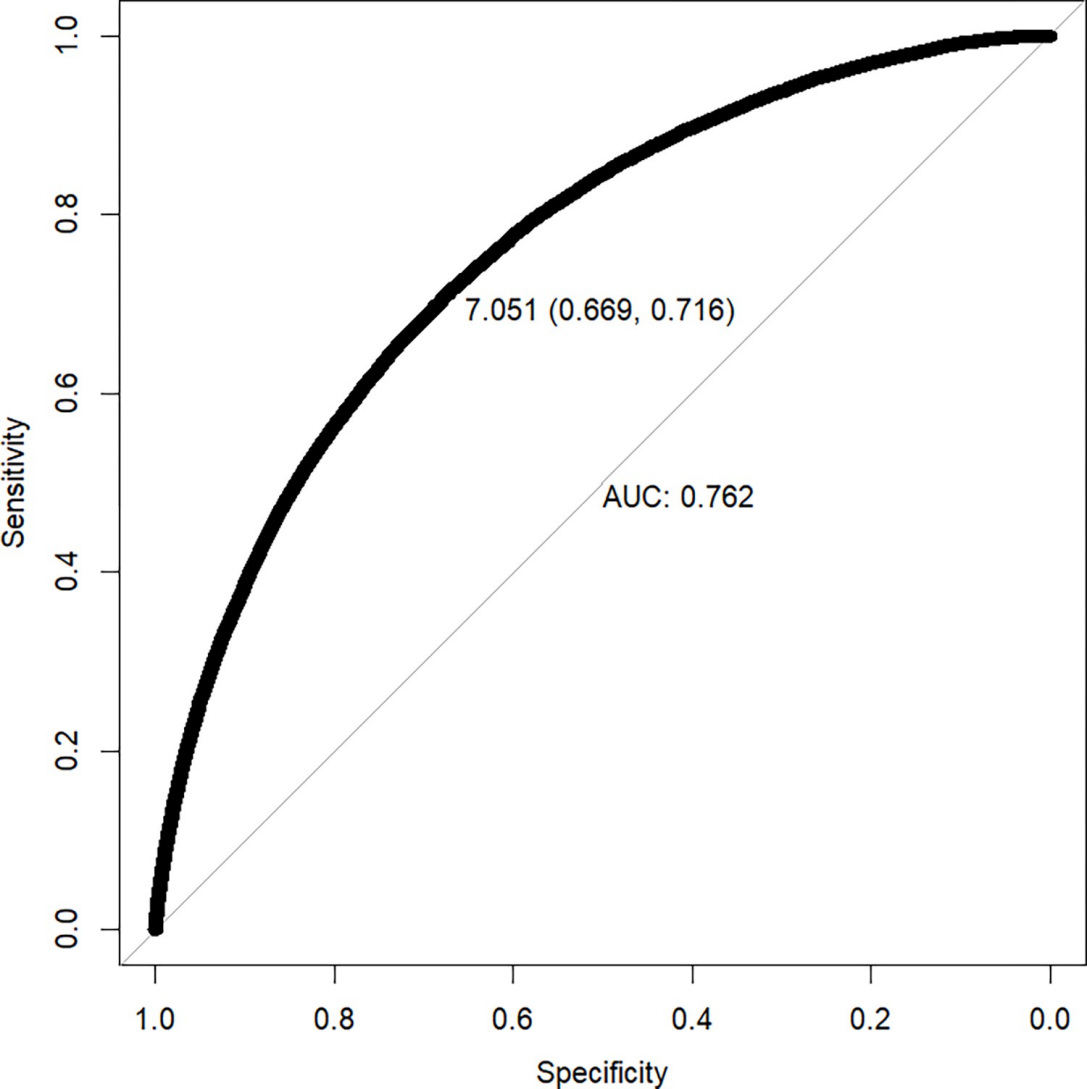
ROC curve and export cutoff productivity. This paper also tries to estimate the export cutoff productivity of pure processing trade firms. The results show that the export cutoff productivity of them is 6.5403, and the corresponding sensitivity, specificity, accuracy and AUC are 0.5769, 0.5522, 0.5528, 0.5935, respectively. Obviously, all the evaluation indicators are very low, and especially the AUC is only 0.5935, lower than 0.6. So, this export threshold does not have an effective classification performance, and the prediction capability is rather low. In addition, this paper further estimates the export cutoff productivity for the whole sample including the pure processing trade firms. The results indicate that the export cutoff productivity for it is 6.8785, and the corresponding sensitivity, specificity, accuracy and AUC are 0.6125, 0.6621, 0.6592, 0.6906, respectively. Compared to the estimation results in Fig 2, including the pure processing trade firms significantly reduces the classification performance of export cutoff productivity, which proves the previous inference in this paper. In view of these facts, it is reasonable to remove the pure processing trade firms from the sample.

Fig 2 shows that the export cutoff productivity based on the whole sample is 7.051, and the corresponding sensitivity and specificity are 0.716 and 0.669, respectively. When all the enterprises satisfying *tfp*_*it*_≥7.051 are hypothesized to be exporters, the ratio of correctly classified exporters (i.e. true positives) to total exporters is 71.6%. When all the enterprises satisfying *tfp*_*it*_<7.051 are hypothesized to be non-exporters, the ratio of correctly classified non-exporters (i.e. true negatives) to total non-exporters is 66.9%. In addition, the accuracy is 0.671, which means that the ratio of correctly classified exporters and non-exporters to total exporters and non-exporters is 67.1%. Furthermore, it is found through calculation that the export cutoff productivity of 7.051 corresponds to a quantile of 65.36%, which indicates that on the whole, the top 34.64% of enterprises may be more likely to become exporters. However, it has to be pointed out that all the evaluation indicators, including sensitivity, specificity, accuracy and AUC, are not very high. The main reason is that in practice, there are many factors that could affect the export participation of enterprises. Although the productivity plays a major role, it is not the only factor. Furthermore, the proportion of true positives’ export to total export is as high as 98.17%. Hence, it can be seen that the incorrectly identified exporting firms are usually those ones with small export volume, which can also be called export cutoff firms. So, from this point of view, the export cutoff productivity of 7.051 still has the excellent classification performance. Hence, it is acceptable that 7.051 is the minimum productivity level required for firms to engage in exporting in general.

#### 4.1.2 Robustness tests

*4*.*1*.*2*.*1 Introducing export decision into estimation of TFP*. Drawing on the practices of Van Biesebroeck [34] and De Loecker [35], this paper introduces firms’ export decision into the intermediate input function to re-estimate the total factor productivity. The purpose of doing this is to take the learning-by-exporting effects into account. On the basis of re-estimating total factor productivity, this paper re-calculates the export cutoff productivity under the full sample. The results are reported in row 1 of Table 3. Table 3 indicates that introducing learning-by-exporting effects has no significant impact on estimation results. First, the export cutoff productivity is almost exactly the same as that in Fig 2. Second, all the evaluation indicators, including AUC, sensitivity, specificity and accuracy, are very close to those in benchmark estimation. So, the benchmark results of this paper are quite robust.

**Table 3:** Robustness tests.

Robustness tests	ECP	AUC	Sensitivity	Specificity	Accuracy	Quantile
Test Ⅰ	7.0506	0.7553	0.6635	0.7176	0.7154	0.7024
Test Ⅱ	7.1612	0.7631	0.7118	0.6759	0.6773	0.6604
Test Ⅲ	7.1939	0.7731	0.7229	0.6823	0.6839	0.6661

Note: ECP = export cutoff productivity (the same below).

*4*.*1*.*2*.*2 Using alternative method to estimate TFP*. Wooldridge [36] proposes an improved approach based on OP and LP methods. This method has two advantages: (1) It can solve the potential identification problem in the first stage. (2) The robust standard errors can be easily obtained even in the presence of both serial correlation and heteroscedasticity. In order to test the robustness of benchmark results more widely, this paper re-calculates the firms’ export cutoff productivity on the basis of re-estimating total factor productivity using Wooldridge’s [36] method (WRDG method). The row 2 of Table 3 presents the corresponding results. It can be found that the results based on WRDG method are also very close to benchmark results in Fig 2.

*4*.*1*.*2*.*3 Taking both WRDG method and learning-by-exporting effects into account*. This part will conduct a further robustness test by taking both WRDG approach and learning-by-exporting effects into account. The row 3 of Table 3 reports the estimation results. It can be seen that the estimation results has no significant changes. Thus, the benchmark results remain robust.

### 4.2 Heterogeneity analysis

#### 4.2.1 Sub-ownership

Table 4 presents the results by ownership. The results indicate that the export cutoff productivity of home firms, including state-owned firms and private firms, is lower, while that of foreign firms, including foreign-invested firms and Hong Kong, Macao and Taiwan-invested firms, is higher. As the state-owned firms can enjoy the tax preference and subsidy from government, they can participate in export trade even if their productivity is lower. The attractiveness of private firms to excellent talents is often limited, which makes their wage cost usually low. In addition, the private firms have the more flexible operation mechanism, and are more sensitive to the market, which makes them have a certain advantage in meeting the individual need of foreign customers. Obviously, this also makes up for the disadvantage of their productivity to a certain extent, and increases the possibility of their exporting. As for the foreign-invested firms, although quite a proportion of them only take China as a production, processing and assembly base to engage in processing trade, the foreign-invested firms that purely engage in processing trade has been excluded from the sample of this paper. Further, the productivity level of foreign-invested firms with ordinary trade is usually higher, which raises the productivity threshold for their exporting. The explanation for Hong Kong, Macao and Taiwan-invested firms is similar to that for foreign-invested firms, and will not be repeated here.

**Table 4:** Sub-ownership results.

Type of firms	ECP	AUC	Sensitivity	Specificity	Accuracy	Quantile
State-owned	6.7570	0.8631	0.8944	0.7037	0.7071	0.6930
Private	6.7266	0.7035	0.7249	0.5739	0.5770	0.5681
Foreign-invested	7.2823	0.6656	0.6644	0.5718	0.5911	0.5226
HK-MO-TW	7.0489	0.6381	0.6288	0.5715	0.5794	0.5441

#### 4.2.2 Sub-industry

Table 5 reports the results by industry. Table 5 indicates that the industries with higher export cutoff productivity are mainly the mining and public utility ones, such as coal mining and washing (06), oil and gas extraction (07), electricity and heat production and supply (44), and gas production and supply (45). In remaining industries, the export thresholds of capital-intensive and technology-intensive industries are relatively high while that of labor-intensive industry is relatively low, which is consistent with our intuition. Specially speaking, the export cutoff productivity of most of labor-intensive industries is below 7, such as food manufacturing (14), textile (17), textile clothing, shoes and hat manufacturing (18), wood processing and wood, bamboo, rattan, palm and grass products (20), and cultural, educational and sporting goods manufacturing (24). Contrary to labor-intensive industries, the export cutoff productivity of most capital-intensive and technology-intensive industries is above 7, and among them, that of quite a few industries is greater than 7.5, such as tobacco products (16), petroleum processing, coking and nuclear fuel processing (25), chemical fiber manufacturing (28), ferrous metal smelting and rolling (32), and electrical machinery and equipment manufacturing (39).

**Table 5:** Sub-industry results.

Code of industry	ECP	AUC	Sensitivity	Specificity	Accuracy	Quantile
06	8.5599	0.9794	0.9667	0.9220	0.9221	0.9203
07	9.3909	0.7934	1.0000	0.6141	0.6237	0.6008
08	7.6798	0.7700	1.0000	0.7400	0.7402	0.7396
09	7.4092	0.8041	0.9091	0.6876	0.6881	0.6865
10	7.2890	0.8235	0.7674	0.7800	0.7797	0.7695
11	8.6563	0.9800	1.0000	0.9800	0.9804	0.9608
13	7.1858	0.7459	0.6755	0.6752	0.6752	0.6682
14	6.9590	0.7693	0.7273	0.6637	0.6663	0.6483
15	7.7886	0.8106	0.6810	0.8099	0.8072	0.7996
16	8.0057	0.7276	1.0000	0.4691	0.4728	0.4667
17	6.9909	0.7487	0.6912	0.6768	0.6776	0.6574
18	6.9154	0.7013	0.6200	0.6751	0.6723	0.6600
19	7.2775	0.7294	0.6142	0.7563	0.7477	0.7343
20	6.8540	0.7165	0.6398	0.6716	0.6709	0.6652
21	6.9845	0.6889	0.5749	0.7074	0.7019	0.6958
22	7.0681	0.8222	0.8069	0.7160	0.7176	0.7067
23	6.7028	0.8236	0.7794	0.7166	0.7176	0.7090
24	6.6446	0.7185	0.6897	0.6117	0.6185	0.5865
25	8.1849	0.7703	0.6849	0.7408	0.7400	0.7342
26	7.3933	0.7996	0.7026	0.7455	0.7433	0.7227
27	7.4058	0.7871	0.7667	0.6747	0.6802	0.6488
28	7.8259	0.8159	0.7314	0.8089	0.8045	0.7781
29	7.1572	0.8048	0.7182	0.7489	0.7469	0.7191
30	6.7461	0.7425	0.7398	0.6407	0.6443	0.6269
31	6.7568	0.7558	0.7629	0.6048	0.6081	0.5972
32	7.8408	0.8653	0.8182	0.7514	0.7530	0.7376
33	7.2873	0.7740	0.8084	0.6040	0.6121	0.5877
34	6.7324	0.7298	0.7374	0.6082	0.6130	0.5954
35	7.0124	0.7930	0.7387	0.7097	0.7113	0.6851
36	7.0576	0.7669	0.7059	0.6949	0.6956	0.6690
37	7.3286	0.8080	0.7193	0.7435	0.7421	0.7167
39	7.5273	0.7616	0.6085	0.7822	0.7719	0.7592
40	7.0822	0.7231	0.7353	0.6070	0.6234	0.5633
41	7.0524	0.7339	0.6546	0.6955	0.6919	0.6650
42	6.9578	0.6954	0.5778	0.7202	0.7116	0.7025
43	7.8159	0.7832	1.0000	0.7291	0.7304	0.7243
44	8.0086	0.8342	0.9231	0.7661	0.7662	0.7658
45	7.9437	0.7118	0.7500	0.8386	0.8384	0.8379

Notes: As the water production and supply industry only includes the non-exporting firms, it is impossible to estimate its export cutoff productivity.

As to the one-to-one correspondence between 2-digit codes and specific industries, see S1 Table.

39 industrial sectors are classified as follows. Firstly, according to Bao and Shao [37], the industries with 2-digit codes of 6–11 are classified as the mining industry, while the industries with 2-digit codes of 44–46 are classified as the public utility industry. Secondly, based on Lu and Dang [38], Dai [39] and Shen et al. [40], the remaining 30 manufacturing industries can be divided into three categories: labor-intensive industry, capital-intensive industry and technology-intensive industry. Among them, the 2-digit codes of labor-intensive industry include 06–11, 13–15, 17–21, 24, 42–46. The 2-digit codes of capital-intensive industry include 16, 22–23, 25–26, 28–34. The 2-digit codes of technology-intensive industry include 27, 35–37, 39–41.

#### 4.2.3 Sub-province

Table 6 presents the results by province. The results show that on the whole, the export threshold of western provinces is relatively high, followed by central provinces and eastern provinces. Specially, the export cutoff productivity of eastern provinces is basically between 6.75 and 7.35, and among them, that of many provinces is below 7, such as Beijing, Liaoning, Zhejiang and Fujian. The export cutoff productivity of central provinces is all above 7, and basically between 7.20 and 7.60. Although the export cutoff productivity of a few western provinces is relatively low, that of most provinces in this region is very high, and basically between 7.40 and 9.00. Among them, the export cutoff productivity of Sichuan, Tibet, Gansu, Ningxia and Inner Mongolia is all greater than 7.50. In fact, the regional heterogeneity of export cutoff productivity here is in line with Melitz’s [2] theoretical expectation. According to Melitz [2], the transportation cost is an important component of export cutoff productivity and is positively correlated with it. Compared to eastern firms, the central and western firms will have to bear more transportation cost to engage in export trade due to their long geographical distance from coastal ports. So, it is not surprising that the export threshold of central and western firms is usually higher.

**Table 6:** Sub-province results.

Province	ECP	AUC	Sensitivity	Specificity	Accuracy	Quantile
Beijing	6.8058	0.8000	0.8129	0.6416	0.6521	0.6136
Tianjin	7.1970	0.8078	0.6742	0.7874	0.7804	0.7587
Hebei	7.3488	0.7558	0.7034	0.6865	0.6869	0.6778
Shanxi	7.5899	0.8024	0.6957	0.7886	0.7877	0.7837
Inner Mongolia	7.5069	0.7349	0.6952	0.6659	0.6664	0.6595
Liaoning	6.8889	0.7910	0.7750	0.6697	0.6735	0.6535
Jilin	7.2217	0.7760	0.7469	0.6795	0.6807	0.6715
Heilongjiang	7.2298	0.8000	0.7086	0.7469	0.7463	0.7394
Shanghai	7.3575	0.7688	0.6411	0.7641	0.7539	0.7306
Jiangsu	7.1482	0.7782	0.7318	0.6844	0.6865	0.6655
Zhejiang	6.7473	0.7718	0.6914	0.7203	0.7184	0.6937
Anhui	7.2232	0.7826	0.6626	0.7683	0.7648	0.7541
Fujian	6.7649	0.7213	0.7371	0.6031	0.6095	0.5867
Jiangxi	7.3715	0.8140	0.6902	0.7937	0.7922	0.7869
Shandong	7.3092	0.7051	0.6836	0.6066	0.6100	0.5936
Henan	7.2654	0.7634	0.7221	0.6755	0.6764	0.6674
Hubei	7.6895	0.8066	0.6630	0.8209	0.8183	0.8129
Hunan	7.0433	0.8078	0.7853	0.6971	0.6984	0.6897
Guangdong	7.2123	0.7642	0.6674	0.7314	0.7281	0.7110
Guangxi	6.8507	0.8023	0.7994	0.6501	0.6551	0.6365
Hainan	7.0801	0.8500	0.8485	0.7474	0.7493	0.7361
Chongqing	7.3963	0.8437	0.7529	0.8090	0.8066	0.7849
Sichuan	7.5014	0.7974	0.7028	0.7525	0.7513	0.7410
Guizhou	6.7296	0.8553	0.9000	0.6891	0.6913	0.6827
Yunnan	7.4464	0.7792	0.6871	0.7851	0.7831	0.7756
Tibet	9.0473	0.9972	1.0000	0.9964	0.9965	0.9941
Shaanxi	7.2960	0.8502	0.7948	0.7771	0.7776	0.7607
Gansu	7.7949	0.9188	0.8298	0.9097	0.9091	0.9045
Qinghai	6.8322	0.7343	0.7692	0.6753	0.6763	0.6687
Ningxia	7.5930	0.7559	0.6977	0.7891	0.7869	0.7782
Xinjiang	7.1238	0.8381	0.8333	0.7498	0.7505	0.7448

Note: Following the traditional geographical division method, this paper divides 31 provinces (municipalities directly under the central government, autonomous regions) of China into three regions: the east, the middle and the west. The east consists of 12 provinces, including Beijing, Tianjin, Hebei, Liaoning, Shanghai, Jiangsu, Zhejiang, Fujian, Shandong, Guangdong, Guangxi and Hainan. The middle consists of 8 provinces, including Shanxi, Jilin, Heilongjiang, Anhui, Jiangxi, Henan, Hubei and Hunan. The west consists of 11 provinces, including Sichuan, Chongqing, Guizhou, Yunnan, Tibet, Shaanxi, Gansu, Qinghai, Ningxia, Xinjiang and Inner Mongolia. The reason why Guangxi is divided into the east is that it is a coastal province.

## 5 Dynamic change of export threshold

**Fig 3:**
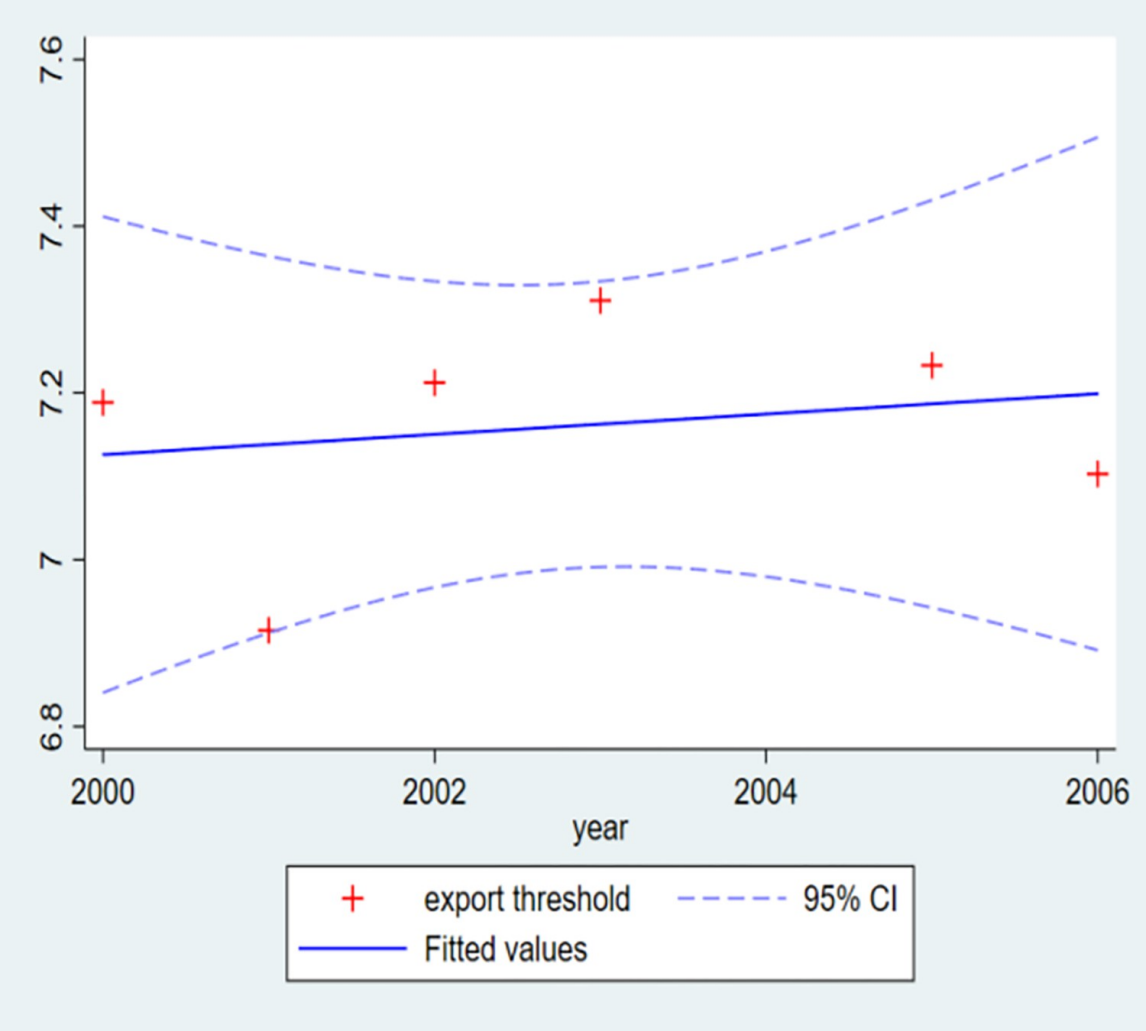
Evolution of export threshold.

Fig 3 indicates that the export threshold decreased in 2001, then increased, reached its peak in 2003, and then declined. So, the evolution of export threshold presents a horizontal S-shape in general, and after China’s accession to the WTO in 2001, an inverted U-shape. The Southeast Asian financial crisis that broke out in1997 sweeps through Thailand, Malaysia, Indonesia, Philippines, Singapore, Japan, South Korea, Hong Kong, Taiwan and Russia, making many countries and regions fall into a severe economic recession. As many countries and regions in crisis are the important trading partners of China, the export environment faced by Chinese enterprises becomes very poor. Although the crisis ended in 1999, it will still take some time for the economies of the harmed countries to recover fully, which caused a higher export threshold in 2000. However, with the weakening of the Southeast Asian financial crisis, the export obstruction faced by Chinese enterprises also decreases, which explains the decline of export threshold in 2001. Although China joined the WTO at the end of 2001, the export threshold does not decline immediately, but keeps rising in the first few years after its entry into the WTO. The possible reasons are as follows. First, the increasing of export enterprises brings about the fierce market competition. The data show that the number of export firms increases year by year during the period 2001–2003, with an average annual growth rate of 21.8%, which indicates that the export competition in the next year is more fierce than that in the previous year. Obviously, if enterprises want to stand out in the more fierce competition, they must improve the production and operation efficiency, thus leading to the continuous rising of export threshold. Second, many WTO members still enjoy the retention clauses within a certain period after China’s accession to the WTO, mainly including European Community, Argentina, Mexico, Poland and Turkey. That is to say, they can continue to impose quantitative restrictions or maintain high tariffs on many products from China during this period. Third, before July 1, 2004, China still implemented the examination and approval system of foreign trade operation right, and thus, many enterprises with export ability still faced the strong institutional constraints. In addition, the main reasons why the export threshold has dropped sharply since reaching its peak in 2003 are as follows. First, the expiration dates of the aforementioned retention clauses are basically between 2004 and 2006. Second, the implementation of registration and record system for foreign trade operators since July 1, 2004 means that many enterprises with export ability are no longer bound. Now, it can be understood that the evolution of export threshold is an inverted U-shape after China’s accession to the WTO.

## 6 Conclusions

Melitz (2003) indicates that only the enterprises whose productivity is above export cutoff productivity can participate in export trade, while the enterprises whose productivity is below it are either eliminated by the market or can only operate in the domestic market. This means that the exporting of heterogeneous enterprises shows a productivity threshold effect. However, due to non-applicability of mainstream threshold estimation methods, the literature on empirical estimation of export cutoff productivity has been lacking for a long time. This not only makes us unable to know the quantitative level of productivity threshold that the exporting of enterprises needs to cross, but also restricts the study on export behavior of enterprises to a certain extent. This paper makes up for this shortcoming. Using two sets of highly disaggregated micro-enterprise data during the period 2000–2006: firm-level production data from CIED and transaction-level trade data of CGAC, this paper firstly estimates the export cutoff productivity of Chinese industrial enterprises under the full sample by applying non-parametric ROC method. Further, it investigates the ownership, industry and region heterogeneity of export cutoff productivity. Then, it examines the dynamic change of export threshold. Specially, the conclusions are as follows.

1. The export cutoff productivity for full sample is 7.051, and the corresponding sensitivity, specificity, accuracy and AUC are 0.716, 0.669, 0.671, 0.762, respectively. The quantile that corresponds to the export threshold is 65.36%, indicating that only the top 34.64% of enterprises have the ability to participate in exporting. In addition, these benchmark results are supported by multiple robustness tests.
2. The export threshold is of significant ownership, industry and region heterogeneity. Specially, the export cutoff productivity of home enterprises is relatively low, while that of foreign enterprises is relatively high. The export thresholds of capital-intensive and technology-intensive industries are relatively high, while that of labor-intensive industry is relatively low. The export threshold of western provinces is relatively high, followed by central provinces and eastern provinces.
3. The evolution of export threshold presents a horizontal S-shape during the whole sample period, and after China’s accession to the WTO in 2001, an inverted U-shape.

## Supporting information

S1 Table A list of 2-digit codes and corresponding industries. (PDF)
