# Supplementary material for: Estimation of export cutoff productivity of Chinese industrial enterprises
Source: PLoS One. 2022 Nov 29;17(11):e0277842. doi: 10.1371/journal.pone.0277842 (PMC10121181; doi:10.1371/journal.pone.0277842)
Supplement: S1 Table — (PDF) [file pone.0277842.s001.pdf]

S1 Table. A list of 2-digit codes and corresponding industries.

Code	Industry name
06	Coal mining and washing
07	Oil and gas extraction
08	Ferrous metal mining
09	Non-ferrous metal mining
10	Non-metal mining
11	Other mining
13	Agricultural and sideline food processing
14	Food manufacturing
15	Beverage manufacturing
16	Tobacco products
17	Textile
18	Textile clothing, shoes and hat manufacturing
19	Leather, fur, feather (down) and their products
20	Wood processing and wood, bamboo, rattan, palm and grass products
21	Furniture manufacturing
22	Paper and its products
23	Printing and reproduction of recording media
24	Cultural, educational and sporting goods manufacturing
25	Petroleum processing, coking and nuclear fuel processing
26	Chemical raw materials and chemical products manufacturing
27	Pharmaceutical manufacturing
28	Chemical fiber manufacturing
29	Rubber products
30	Plastic products
31	Non-metallic mineral products
32	Ferrous metal smelting and rolling
33	Non-ferrous metal smelting and rolling
34	Metal products
35	General equipment manufacturing
36	Special equipment manufacturing
37	Transportation equipment manufacturing
39	Electrical machinery and equipment manufacturing
40	Communication equipment, computer and other electronic equipment manufacturing
41	Instrument and culture, office machinery manufacturing
42	Arts and crafts and other manufacturing
43	Waste resources and waste materials recycling and processing
44	Electricity and heat production and supply
45	Gas production and supply
46	Water production and supply
